# Preserving the Impossible: Conservation of Soft-Sediment Hominin Footprint Sites and Strategies for Three-Dimensional Digital Data Capture

**DOI:** 10.1371/journal.pone.0060755

**Published:** 2013-04-17

**Authors:** Matthew R. Bennett, Peter Falkingham, Sarita A. Morse, Karl Bates, Robin H. Crompton

**Affiliations:** 1 School of Applied Sciences, Bournemouth University, Talbot Campus, Fern Barrow, Poole, Dorset, United Kingdom; 2 Structures and Motion Laboratory, Department of Comparative Biomedical Sciences, Royal Veterinary College, London, United Kingdom; 3 Institute of Aging and Chronic Disease, University of Liverpool, Liverpool, Merseyside, United Kingdom; 4 Division of Biology and Medicine, Department of Ecology and Evolutionary Biology, Brown University, Providence, Rhode Island, United States of America; University of Florence, Italy

## Abstract

Human footprints provide some of the most publically emotive and tangible evidence of our ancestors. To the scientific community they provide evidence of stature, presence, behaviour and in the case of early hominins potential evidence with respect to the evolution of gait. While rare in the geological record the number of footprint sites has increased in recent years along with the analytical tools available for their study. Many of these sites are at risk from rapid erosion, including the Ileret footprints in northern Kenya which are second only in age to those at Laetoli (Tanzania). Unlithified, soft-sediment footprint sites such these pose a significant geoconservation challenge. In the first part of this paper conservation and preservation options are explored leading to the conclusion that to ‘record and digitally rescue’ provides the only viable approach. Key to such strategies is the increasing availability of three-dimensional data capture either via optical laser scanning and/or digital photogrammetry. Within the discipline there is a developing schism between those that favour one approach over the other and a requirement from geoconservationists and the scientific community for some form of objective appraisal of these alternatives is necessary. Consequently in the second part of this paper we evaluate these alternative approaches and the role they can play in a ‘record and digitally rescue’ conservation strategy. Using modern footprint data, digital models created via optical laser scanning are compared to those generated by state-of-the-art photogrammetry. Both methods give comparable although subtly different results. This data is evaluated alongside a review of field deployment issues to provide guidance to the community with respect to the factors which need to be considered in digital conservation of human/hominin footprints.

## Introduction

Within the geological record fossilised human footprints have been found preserved in a range of depositional environments providing information about both the presence and palaeoenvironment of our ancestors, and in a few cases that pre-date anatomically modern *Homo sapiens* informing us about the evolution of human gait (e.g., [1]–[2]). The number of footprints sites now documented in the literature has grown dramatically over the last forty years, especially those sites of Holocene or Late Pleistocene age [3], [4]. While some of these footprints are preserved in partially lithified volcanic ash such as those found at Laetoli in northern Tanzania (3.66 M years) [5] or those from Jeju Island in Korea (15 K years) [6], most are preserved in unlithified, fine-grained silt and fine sand such as those at Ileret in northern Kenya (1.5 M years) [7]. In some notable cases of Holocene age, prints are exposed by coastal erosion and then destroyed (e.g., [8]–[10]). The conservation of these soft-sediment footprint sites, especially for sites of palaeoanthropological significance like that at Ileret is challenging and a subject which has received little attention to date with the exception of the debate surrounding the conservation of the Laetoli prints [11]–[15].

In parallel with this progressive increase in the number of known human footprint sites has been the increasing availability of field strategies with which to capture and record fossil prints in three dimensions (e.g., [15]–[20]) and crucially to analyses this data objectively (e.g., [2], [7], [21]). In part this has been driven by the increased use of digital data in the study of dinosaur footprints (e.g., [16]–[21]). Traditional solutions of manual photogrammetry deployed at Laetoli [22], have been replaced by the increasing use of digital photogrammetry [20], [23], [3]. A range of optical laser scanning methods have also been deployed at footprint sites from long range Light Detection and Ranging (LiDAR) imaging [17], to close quarter and high resolution scanning [7]. The increased availability of methods with which to capture the three-dimensional surface of a print and to subsequently output it via three-dimensional printing or rapid proto-typing technology [24] provides a viable conservation and preservation strategy with which to preserve fragile and eroding footprint sites, but equally poses challenges with respect to the most appropriate digitising technique to adopt [25]. In this context the aim of this paper is two-fold: (1) to explore the geoconservation of soft-sediment human/hominin footprint sites; and (2) in light of this explore the role that digital conservation and specifically evaluate two alternative approaches – photogrammetry and optical laser scanning - with which to do so.

## Part One: Geoconservation of Human Footprint Sites

### Spectrum of Sites

**Figure 1:**
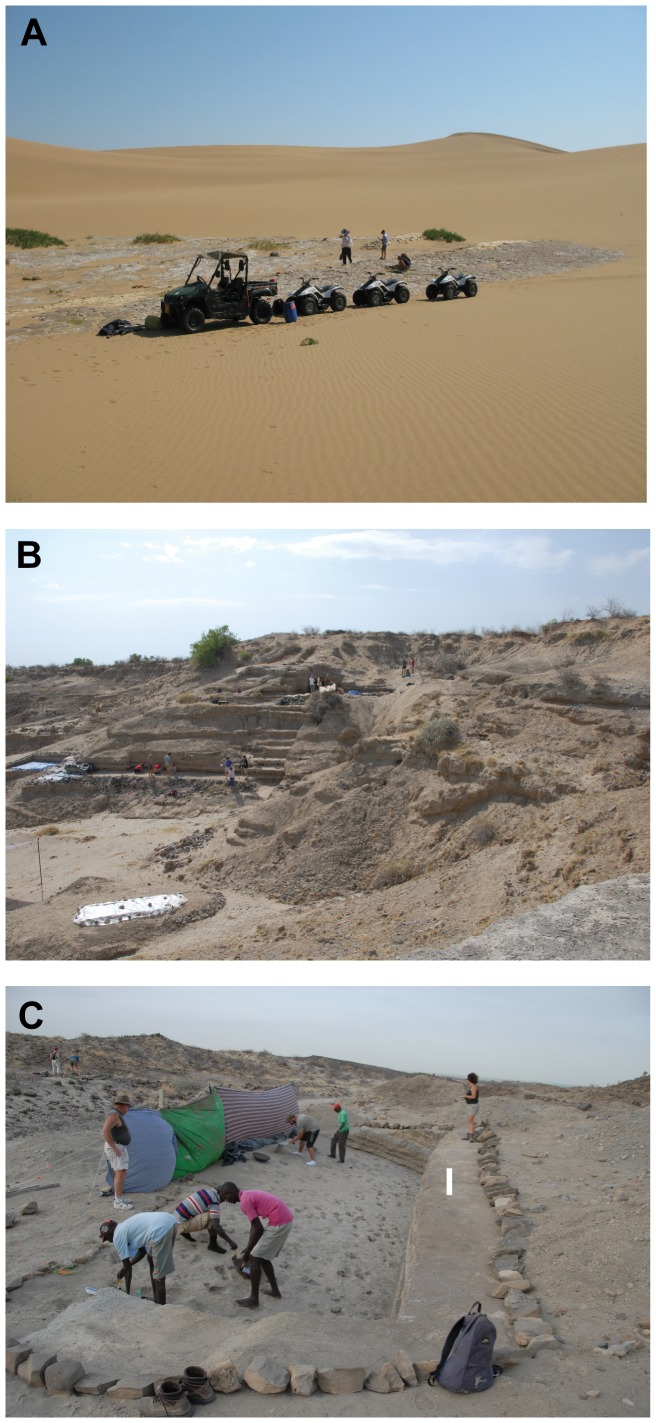
Examples of soft-sediment footprint sites. **A.** Footprint site south of Walvis Bay, Namibia. Migration of active dunes across silt surfaces reveals a range of Holocene footprints. **B.** FwJj14E footprint site close to the village of Ileret in northern Kenya. The prints occur at multiple levels within the eroding silt bluff. Note the rock armour introduced below the lower surface to combat seasonal storm run-off and erosion. **C.** GaJi10 footprint site south of Koobi Fora in northern Kenya. The site is located adjacent to the bed of a seasonally active river and as shown the footprint rich beds dip into the slope away from the thalweg of the channel.

While all historical/archaeological/fossil human footprints are of note and attract scientific and public interest, some are clearly of more significance in a palaeoanthrpological context than others. Human footprint sites are preserved in a range of different depositional environments which now outcrop, and are exposed, in a variety of geomorphological settings [3] and as such are exposed to different levels of preservation risk. For example, the mid-Holocene footprints of the Sefton Coast in northern England [8], originally formed in coastal dune slacks during a period of lower sea level, are now exposed by storm events which draw-down protective beaches to expose the underlying ichnologically-rich silt beds. Sections of this imprinted surface are exposed for relatively short intervals before being lost to coastal erosion. In Namibia, footprints (Fig. 1) [26] are exposed on terrace surfaces formed of fluvial over-bank flood deposits which were desiccated, imprinted and subsequently buried by mobile sand dunes and are exposed periodically as dunes migrate over the surface. These prints are quickly eroded and deflated when the salt hardened silt is disturbed either naturally or increasingly by tourists and by recreational vehicles exploring the dune fields. Sites like these are of local archaeological significance and their true scientific value is perhaps more limited, although one should not underestimate their importance to local populations and heritage tourism.

There are sites of greater scientific value in terms of their ability to aid in the understanding of human evolution, which are also arguably aided by the continued operation of natural geomorphological process. For example, at Nahoon in South Africa a trail of human prints is preserved in aeolinites dated to 124±4 k BP and are potentially amongst the oldest examples of *Homo sapiens* prints in Africa, although the more equivocal Langebaan prints provide a close rival for this title [27], as do the recently discovered prints close to Lake Natron in Tanzania [28]. The Nahoon prints were first observed in 1964 within an overhang which then collapsed with two prints rescued and transported to the local East London Museum [27]. The original site, on the sea cliffs at Nahoon, is maintained as tourist attraction and excavation reserve, and is marked by a footprint shaped visitors centre. One could argue that continued coastal erosion and cliff collapse has the potential to reveal new prints in time, despite the risk that some may be lost during that process. The conservation strategies relevant to each of these sites is potentially different but in all cases allowing natural process to continue – dune migration and coastal erosion – is positive since in both cases new prints are exposed for study.

This type of approach sits in contrast with that adopted at Laetoli in northern Tanzania which is probably the most valuable currently known palaeoanthropological footprint site, in the world. The prints were first excavated in 1978–79 and are preserved in volcanic ash, lithified to varying amounts via a secondary deposit of calcite, and were carefully documented at the time using the latest technology, including hardcopy vertical photogrammetry, and carefully casting with a variety of media [29], [30]. These casts and moulds have been used widely to supply teaching models around the world and used extensively in research (e.g., [2], [31]). The site was originally re-buried with backfill which was unfortunately rich in acacia seed, the growth of which provided a clear threat to the conservation of the site leading to its re-excavation in the 1990s [12]–[15]. Demas and Agnew [12] documented the decision making process which led to the re-burial of the site in a controlled manner involving the use of a range of geo-membranes acting as barrier layers to prevent a recurrence of the root problem. The solution while effective [13] remains an area of continual tension both for the scientific community who are denied access and because of the inability both locally and nationally to derive tourist and cultural value from the site. It is worth nothing that an exhibition at Olduvai Museum including replicas of some of the original casts provides compensation in part for the local community and international tourists. A range of alternative plans have been mooted in recent years including the complete excavation and removal to some form of either local or national museum [15]. The strategy here is clear, to preserve at all costs a finite resource, something which is aided by the fact that although fragile, the substrate is partially lithified.

In effect there is a spectrum of sites from those of relatively low scientific value, often with high print numbers, which are located in sites that are threatened continually by natural processes but are in turn dependent to some degree on natural process for exposure (e.g., Sefton Coast or Namibia), via those at Nahoon or Langabaan which are of greater scientific significance and are more limited in extent, to those at the other extreme such as Laetoli which are of considerable scientific importance, limited in number, but are preserved in a comparatively firm substrate and are consequently threatened less by natural processes. The Ileret footprints in northern Kenya [7] sit uncomfortably within this spectrum, being arguably of considerable scientific importance, modest in number but preserved in what is highly erodible, unlithified sediment and consequently form an interesting case study.

### Ileret Footprints: a Case Study

The prints located close to the Kenyan village of Ileret (Site: FwJj14E) [7] have been dated to 1.5 million years old and are (Fig. 1) therefore the second oldest footprint site in the world. The site is preserved in unlithified, fine-grained silt and sand deposited in low energy fan deltas at the margins of playa lakes on the floor of the Turkana rift valley [32]. The Ileret site itself consists of an eroding bluff 5 m high exposing horizontally bedded, early Pleistocene layers which are inter-bedded with three volcanic tuffs used for dating purposes. Footprints occur on several bedding planes and the site is actively eroding via gullies and slope wash, during periodic storm run-off events (Fig. 1). Bennett et al. [7] also reported on the re-excavation of a slightly younger footprint site (Site: GaJi10) 45 km to the south close to Koobi Fora, consisting of a short hominin trail, first reported by Behrensmeyer and Laporte in 1981 [33]. In contrast to Ileret the fine sand and silt beds are inclined, due to normal faulting and associated block rotation, at approximately 32° to the northwest. The footprint site is located at valley floor level on the northern flank of a dry river valley (Fig. 1). Excavation of the footprint surface into the valley side is limited by the increased over-burden and the excavated site is at risk from flooding and erosion during storm events. The original site excavated by Behrensmeyer and Laporte [33] was re-buried with backfill material and in places a layer of canvas or plastic to act as a marker horizon. In terms of conservation this has proved adequate, because on re-excavation almost 30 years later the site was largely preserved undamaged apart from the loss of one of the hominin footprints due to erosion along the axis of the valley floor. The re-excavated prints proved to be far superior in quality to the fibreglass cast of the site held at the National Museums of Kenya and dating from the original excavation [7].

The key conservation threats at both of these two Kenyan footprint sites can be summarised as: (1) sediment weathering and gravity driven slope failure; (2) lateral fluvial erosion and/or rain induced gullying during wet seasons; (3) bio-erosion due to roots, animal burrows and the passage of grazing livestock; (4) unlawful excavation; (5) inappropriate exploitation by indigenous populations, for example removing valuable assets such as plastic sheeting or accidental, curiosity driven damage; (6) damage during repeated re-excavation during successive field seasons; and (7) break-up of the sediment surfaces due to changes in sediment moisture content (causing swelling or desiccation), thermal expansion/contraction and vertical unloading all of which can be caused by changes to overburden volume, surface run-off and hydrology geology during excavation of benches and introduction of plastic sheeting and other impermeable membranes by excavators. Of these the most important are probably changes to the sediment moisture content and natural erosional process in semi-arid environments.

Demas and Agnew [14] provided a framework in which to consider the conservation management strategy at Laetoli, namely a review of values, benefits and stakeholders. They also argued that the key to success is joint rather than consensual decision-making on the basis that no one solution will necessarily please all parties. Using this framework it is possible to suggest that the key issues at Ileret and Koobi Fora are:

- Values. Demas and Agnew [14] see this as the value of the site’s research contribution both at the time of discovery, currently and its future potential. There is also the symbolic and spiritual value of sites like this; as stated by Demas and Agnew ([14], p.67) ‘footprints offer a unifying and potent symbol of our species and our beginnings’. The Kenyan sites represent the second oldest footprint localities in the World. Though Laetoli is older, the Kenyan sites appear to represent the feet of *Homo egaster/erectus* [7] and therefore are on the other side of the *Australopithecus* to *Homo* divide - one of the most important stages in human evolution, representing the transition to more open habitats, endurance walking and running and consequently potentially greater migration [34]. The assumption has been made to date that the prints discovered at both sites in Kenya are likely to be derived from the same species of track maker, but the fact that at least three hominin species were roaming the landscape at this time [34] all of which might have left a footprint record, raises a tantalising possibility.
- Benefits. The benefits to the scientific community and the quest to understand human origins and evolution are considerable and taken as read here. The potential benefits to the local community are provided by the revenue introduced to the local economy by foreign excavators and potentially via heritage tourism, although the remote nature of the sites, and the lack of indigenous wildlife which has been hunted to near extinction, may be a serious limitation to this and the tribal/political instability of the region close to the Ethiopian and Somalian borders makes it an unlikely tourist destination at the present.
- Stakeholders. The stakeholders are multiple but include the original excavators, the current excavators and permit holders, the scientific community at large, the National Museums of Kenya responsible for issuing the excavation/collection permits, the local community and tribal elders, as well as the regional and national community. As the site lies just to the north of the Sibiloi National Park all of these stakeholders have a potential voice in the outcome; it is not something that should or can therefore be determined simply by the original excavators and current permit holders, although one could argue they have a responsibility to take the lead.

To date the conservation options for the Kenyan sites have yet to be explored in full although they have been scoped tentatively [35]. We recognise three basic options: (1) on-site conservation; (2) off-site conservation; and (3) do nothing. Options for on-site preservation include a range of possibilities from exposed display to some form of shelter. Unprotected exposure is not an option since the surface would be uncovered and quickly erode under seasonal rain, a process enhanced by seasonal desiccation cracking and individual grain spalling. Surface hardening with resin might prevent this but it is hard to envisage stabilisation of the whole surface without causing changes to the moisture content and dynamics of underlying or adjacent beds leading to structural failure of the hardened slab. Construction of a shelter to protect the footprint surface from the elements, such as that adopted at the Acahualinca footprint site in Nicaragua [36] along with control of surface run-off may provide an option. Agnew [37] provides a useful review of the factors to be considered when opting for some form of shelter at archaeological sites and suggests that solutions require high capital investment, on-going maintenance and a commitment from the local community and the availability of tourists both scientific and general. Whether the site is of sufficient scientific ‘value’ to merit this investment and whether investment can be found are pertinent questions. Engagement from local stakeholders would be vital and with the absence of passing tourists the site is unlikely to provide sufficient economic return for the local community in order to get them to invest time and energy in site stewardship, although clearly this could be explored.

Covered display would provide protection from rainfall and slope run-off during storm events, but would require maintenance since it would naturally channel water which increases the risk. The source of the capital investment and continued maintenance is also an issue. The lack of passing tourists also precludes the likelihood of significant tourist revenue for the local community. In the case of GaJi10 one needs to control storm flow from the adjacent river bed since any excavation void would be lower than the adjacent river floor. The challenges of designing a structure and drainage control for both GaJi10 and FwJj14 are considerable, although not insurmountable given financial investment and on-going maintenance. The value of doing so is however questionable given the lack of tourist facilities despite the field stations of the Turkana Basin Institute and National Museum of Kenya at Koobi Fora. The final on-site option is buried display in which the site is buried for the long-term using the lessons gained from Laetoli [15] coupled with either an on-site display or local museum. It is worth pointing out that burial has worked well at GaJi10, but is unlikely to be so successful at FwJj14E since the prints are part of a naturally eroding bluff.

Off-site options involve the removal of individual blocks or sections of a surface to either a local or regional/national museum for display. Both the South African footprint sites of Langebaan and Nahoon have been removed as blocks and are now stored in museums [27], for example although their removal has been facilitated by the material being heavily lithified. One of the footprints from Cuatro Ciénegas is stored in the Museum of the Desert at Saltio, but its provenance with the actual footprint site which has only recently been re-discovered is unclear and demonstrates some of the risk of breaking the link between a museum specimen and the original site [38], and the importance of thorough documentation when excavating. Given that the surface is unlithified and friable the ability to remove large blocks and transport them from the remote site is questionable given the transport infrastructure available. Footprints also represent additional challenges to curation compared with body fossils in that track sites tend to require large areas for display and/or storage. Excavation and collection of individual prints from a larger site is undesirable for the reasons noted above, but collecting and curating an entire or even partial track surface is often beyond the means of most museums.

The final option is to do-nothing but put in place an on-going monitoring and recording programme consistent with, for example, approaches within rescue archaeology [39]. The argument here is that while each footprint is highly valuable the very act of slope erosion will reveal more of the imprinted surfaces and that their continual erosion is a positive action provided that the data is captured as it is exposed but before it is eroded. One of the limitations to current excavation is the amount of overburden. The depositional environment is extensive and the concentration of hominins in water-rich areas is likely to be such that the probability of more prints being found laterally or at adjacent sites is high. This appears to be the case at Ileret since subsequent excavations to those completed in 2009 has revealed additional prints. Such a strategy acknowledges the futility of trying to preserve soft-sediment sites such as this and changes the emphasis from one of preservation of the physical prints to the more abstract conservation of scientific data and its public dissemination. The key is how the data is recorded and then shared throughout the scientific community for use by all, and how this resource is used to drive in country tourism activity.

### Rescue Archaeology and Hominin Footprint Sites

**Figure 2:**
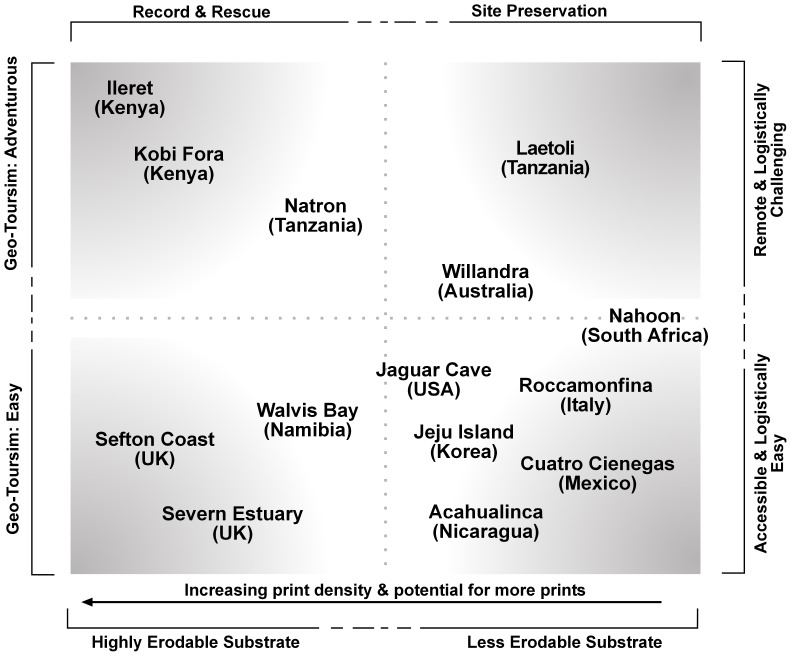
Matrix of variables relevant to the conservation of hominin/human footprint sites with particular emphasis on soft-sediment sites. The horizontal continuum at the top is between strategies based on ‘record and rescue’ versus those based on site preservation either via burial such as Laetoli or via some form of conserved display as is the case at Acahualinca in Nicaragua.

One could argue that all the above footprint sites discussed so far fall along a continuum with site preservation at one end of the spectrum and ‘record and digital rescue’ at the other (Fig. 2). This is irrespective of their ‘value’ defined either by the current scientific community or by the public at large. Within this there are a range of variables which need to be considered from logistical access, heritage tourism potential, print density and potential for more prints, through to the geological factors which control the risk of substrate erosion.

There is a parallel here with approaches to geoconservation developed in the UK during the late 1980s [40]. As part of a systematic review of geological Sites of Special Scientific Interest in the UK a strategy for their subsequent management was developed [41] which was based on their classification into one of two types:

1. Exposure sites, such a coastal cliffs, river cliffs or quarry exposures where the management aim was to maintain the *exposure* which was assumed to be essentially limitless. For example, where there was an extensive outcrop of a particular fossil-rich horizon the aim was to maintain access to that outcrop via fresh or maintained exposures. In these cases maintaining active erosion of coastal or fluvial cliffs, faces within worked quarry, or in the case of soft-sediment sites preserving ‘green-field’ areas where scientific excavation, if not public viewing, could occur in the future was the driving aim of conservation management; a clear strategy to maintain access and in some cases even celebrating erosion and quarrying!
2. Integrity sites, where the aim was to preserve a resource of more limited extent or number. For example a defined area of mine tailings where exotic minerals could be found recovered or a lagerstätten of limited extent where the aim is to protect every last piece of a very small resource.

This framework provides an interesting perspective on the Ileret case study and similar sites since one might at first principal assume that they are integrity sites since there are few footprints and every one must therefore be preserved. However, at sites like this where there are extensive areas with the *potential* to contain prints, in this case extensive areas of a fine-grained fan delta and evidence of human congregation, then one could argue that there is a high probability that prints are more ubiquitous than previously thought, but at this point are just undiscovered. This is confirmed by the fact that extensive exposures of animal prints have been found laterally to GaJi10 and further hominin prints lateral to FwJj14E. The limitation to study is actually that they are buried deeply by overburden, making continued erosion a positive factor since it will reveal new prints for study. This is similar to the UK coastal footprint sites in the Severn Estuary [42] or on the Sefton Coast [9] where continual coastal erosion exposes new prints for study which cannot be preserved only recorded and rescued. The key difference here is that unlike a typical UK coastal fossil site the value to the palaeo-anthropological community of a site such as Ileret is much greater as is the symbolic and cultural value to both indigenous and foreign populations. In both cases we would argue that it is the quality of the record and digital rescue approach used that is key and how this is subsequently made publically available to all stakeholders not just the small group of scientist that hold the current excavation permit.

Record and digitally rescue is therefore about developing a conservation strategy that is able to create a virtual representation of the site, along with the sedimentary and palaeoenvironmental information it contains that will provide a timeless resources for future scientific study that is accessible to all and crucially a platform for public engagement both at and beyond the site. It is also about timely and continual intervention to make sure data is not lost and involving the local community in that process is potentially one approach. A more radical strategy of this sort is made possible by the increasing availability and accuracy of methods of capturing three-dimensional data thereby increasing the accuracy and sophistication of record and rescue in the context of human footprints. In this context it is essential that the scientific community has a clear understanding of the different approaches and there relative merits.

## Part Two: Digital Data

If, as argued above, ‘rescue and digitally record’ is the only viable option for many human footprint sites such as that at Ileret, then the importance of digital capture as part of the recording programme is critical. The technology exists to compile accurate and reliable three-dimensional data allowing both quantitative analyses in the present and future as well as reproductions to be created for display and public engagement. The challenge that exists at the moment is to optimise data collection and for practitioners to understand the relative merits of both photogrammetry [20] and optical laser scanning (e.g., [7]).

The construction of digital elevation models using photogrammetric methods is well established and manual photogrammetry using vertical stereo-pairs taken with a custom built tripod was used at Laetoli [29] and during the original Koobi Fora excavation (GaJi10), although in the latter case only used to derive stereo-pairs for visualisation [33]. Breithaupt et al. [16] document a range of different approaches to collecting photogrammetrical data for dinosaur tracksites in Wyoming, a theme that has been developed by others with some limited method comparison being undertaken (e.g., [25]). The advent of digital photography and the increasing availability of soft-copy photogrammetrical software options has increased the flexibility, accuracy and precision of digital elevation models which require multiple oblique images of a subject enhancing the ease of deployment [20]. In parallel the development of a range of survey tools based around Light Detection and Range (LiDAR) has provided an alternative approach [17]. The systems range from long-distance scanning devices which collect point cloud data from closely spaced intervals, to close quarter scanners which collect data over a continuous stripe. While long-range LiDAR has been used now to good effect at a number of dinosaur trackway sites (e.g., [16]–[18]) its application to human footprints sites has been more limited, possibly due to the relatively low point density spacing for most of these long-range systems and because the sties tend to smaller with fewer tracks. Bennett et al. [7] used close-quarter optical laser scanning at the disputed Valesequllio footprint site in Mexico, developing the necessary equipment to allow field deployment [24] and the same data was used to demonstrate that these marks were in fact not human footprints at all [43]. A revised version of this approach was used at both the Ileret and Koobi Fora footprint sites [7]. Laboratory based scans of casts of the Laetoli footprints have been used widely in a number of analyses [1], [2], [31]. While a number of studies have explored both approaches of recording prints and integrated both types of data sources critical comparison remains elusive [25] and those faced with a rescue and record situation have little guidance. This is not just a question of the accuracy and precision of the models produced, but also the wider context associated with the field deployment of both techniques.

In practice the accuracy and precision of both methods is dependent in part on ambient environmental conditions at the time of capture and the equipment and software used and as such there are a plethora of variables which constantly change as technology improves. Issues of deployment however remain irrespective of equipment and software. Our aim here is to provide guidance to those adopting a rescue and record approach by first using a simple experimental set-up to examine the potential data quality issues and secondly to place this in the context of our experience in deploying both methods in the field.

## Methods

Two simple experimental sets-ups were used in this analysis, one lab-based and one field-based. The lab-based experiment involved the application of both photogrammetry and optical laser scanning to a series of concrete and plaster footprints. A series of four trays were filled with various mixes of sand, cement and plaster to replicate a range of substrate conditions and the same adult male foot imprinted on all four trays and allowed to set. A ruler was fixed to each print along with four wooden 1 cm cubes. Working on the laboratory floor in natural day light each print was scanned once using a tripod mounted optical laser scanner (Vi900 Konica-Minolta) and photographed using a high quality (Nikon D200, 10 mp) camera from multiple elevations and angles creating a minimum of twenty oblique photographs per print. In the field a trail of human footprints was created at low tide, on sandy beach at New Brighton on Merseyside (Lat. 53° 26.3″ N, Long. 3° 2.6″ W). This trail of fifteen footprints was made under normal walking, in a straight line, by a male subject of medium build (height: 176 cm; weight: 69 Kg). Ten contiguous prints were selected from the middle part of the trail and each print was bracketed by four wooden cubes; one either side of a print’s heel and one either side of the toes. The wooden cubes used where 1 cm in size, painted fluorescent-orange, and attached to 6 inch nails with epoxy resin. Each cube was inserted flush with the beach using the nail to prevent movement. The dimensions between each cube were noted using a metal tape. Each print was scanned using an optical laser scanner (Vi900 Konica-Minolta), mounted on a carbon fibre rig and shielded from sunlight. In all scans at least three of the four cubes were included in the visible frame. Once completed each print was then photographed between 20 and 40 times using a Canon Powershot G11 (10 mp) from a range of different angles and elevations to provide material with which to generate photogrammetrical elevation models.

In all cases the optical laser scans were captured in Konica-Minolta Polygon Editing Tool and either output as a cdm file for subsequently manipulation within Rapidform 2006 or output as XYZ point clouds in asc format. No holes were filled or singularities deleted and files were presented as captured by the scanner. Photogrammetrical models were produced using the freely available open source software bundler [44]–[45] and PMVS/CMVS [46]–[47] as in the workflow described and demonstrated by Falkingham [48]. Photogrammetrical models were also produced for the concrete prints using alternative commercial proprietary software (Agrisoft PhotoScan), to illustrate the range of results obtained from the same photographs. The point cloud data was imported in to Foot Processor, a piece of bespoke freeware that allows rapid visual editing of XYZ data files in order to: (1) rectify prints to the orthogonal plane; (2) rotate prints into a consistent longitudinal orientation; (3) mirror left into right prints to allow comparison of all prints within a trail; (4) crop extraneous material from the margins of a print; and (5) contour plot, place landmarks and measure inter-landmark distances.

## Results

### Experiments

We recognise two broad stakeholder groups, the scientific community and the general public inclusive of the landowners and local population. The former group need accurate digital data with which to drive intra- and inter-site comparisons and quantitative analysis, while the latter need three-dimensional data with which to accurately re-produce the prints, either physically or digitally for public engagement purposes. Both are ultimately interested in the accuracy and precision of the digital elevation models.

**Figure 3:**
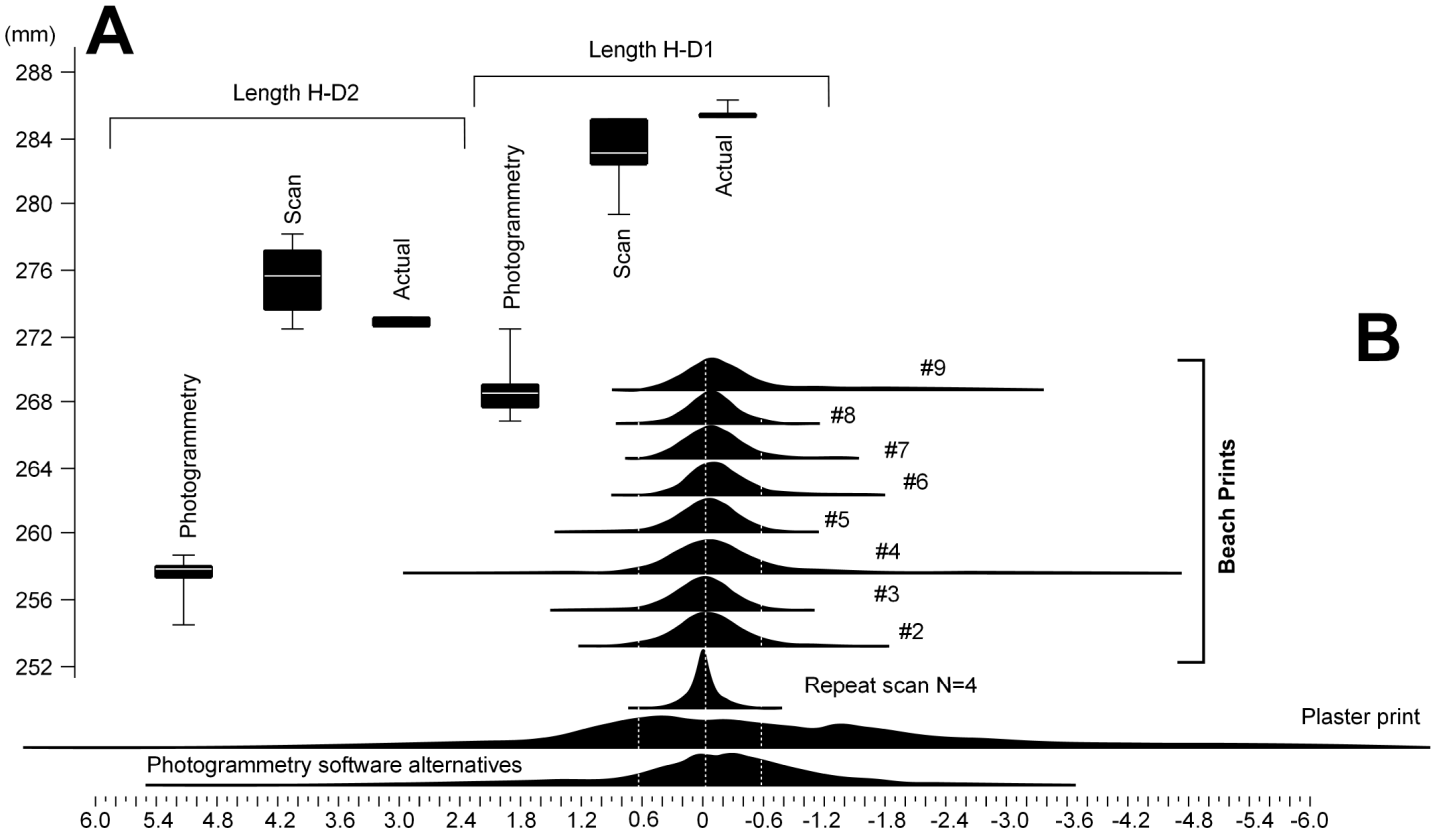
Comparison of photogrammetry and optical laser scanning methods. **A.** Box plot of length dimensions taken from one of the concrete prints, showing the underestimate of length provided by the photo-model. **B.** Shell to shell deviations between co-registered scans for specific prints. The frequency distribution shows the range of deviations both positive and negative. The broader the area of distribution the more divergent the scan shell and the photo-model shell are. Co-registration and shell deviations were undertaken and calculated within Rapidform 2006.

At the simplest level of analysis, scientists need to be able to place landmarks and record inter-landmark distance to gather basic geometric data such as print length or width. Photogrammetric models must be scaled either during construction or subsequently in a three-dimensional editing tool such a Meshlab. There are various ways this can be achieved such as including a three-dimensional element of known dimension, or visually from a scale bar included within an orthorectified image draped over a point cloud or polygonal surface. The accuracy of any linear measurements derived subsequently from the elevation model is therefore dependent on the precision with which this scaling is undertaken. In contrast this is not an issue with most optical laser scanners which locate points in real coordinate space and provided that they are calibrated regularly operate typically in fractions of a millimetre. To illustrate the issue the photogrammetrical models of the concrete prints were scaled in Meshlab independently of the scans and simple linear length dimensions, heel to first toe and heel to second toe, were recorded to one decimal place in Rapidform 2006 for both the scan and photo-model, as well as on actual concrete prints using vernier callipers. The photo-models underestimated the distances significantly in all four cases as illustrated in Figure 3A emphasizing the potential inaccuracy introduced due to scaling errors. One of the principal problems with including geometrical objects within scans to assist with scaling, for example the wooden cubes used here, is that sharp edges are not necessarily well produced in the photo-models since most of the algorithms used are fine tuned to smooth surfaces since these are most appropriate to the majority of print surface. Scaling by photo-images depends on accurate visualisation of the scale bars. This calibration needs to be done for each model and the potential for inter-model variation is present.

For analytical methods based on ‘whole foot’ statistical techniques that compare depth pixels such as pedobarographic Statistical Parametric Mapping (pSPM) [2], [49] this is less of an issue since individual prints are co-registered against one another pixel by pixel and provided they approximate in size initially, the least squares matching algorithms used ensure effective relative scaling of the prints.

**Figure 4:**
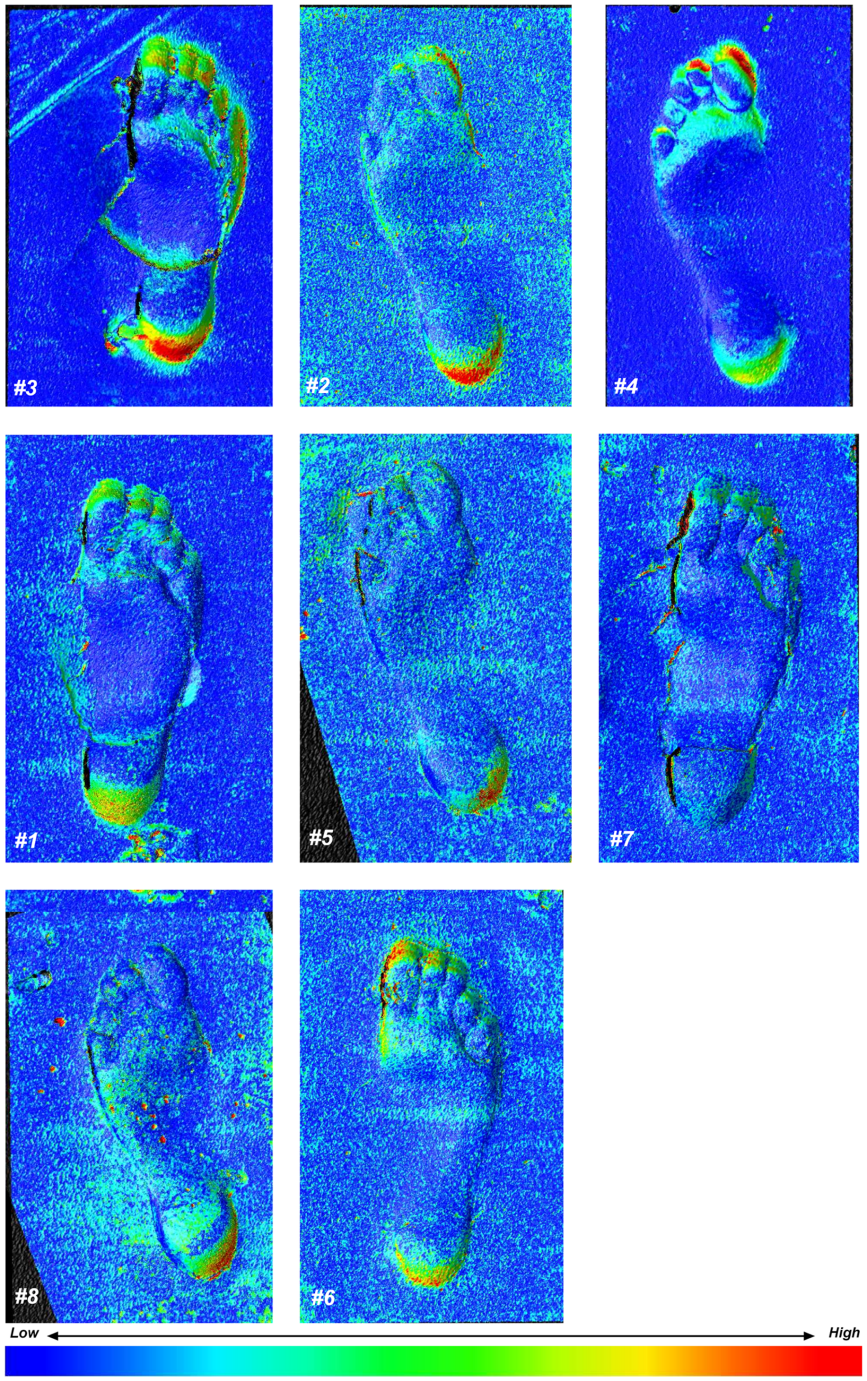
For the eight beach prints the two models/shells for the scanner and one for the photo-model were co-registered in Rapidform 2006 and the maximum model thickness or deviation was calculated and attached as vertex colour map to the combined model. Warm colours indicate maximum thickness or deviation.

**Figure 5:**
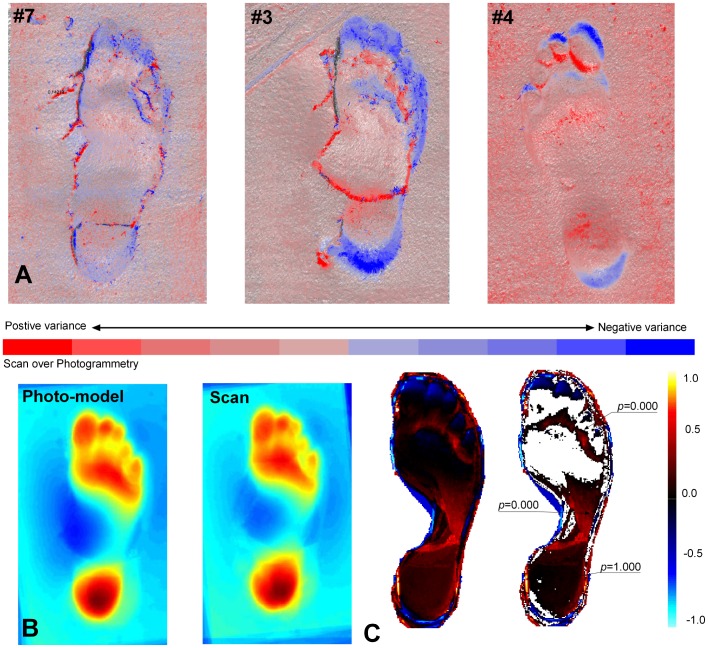
Results showing statistical comparison of photogrammetry and optical laser scanning methods applied to beach prints. A. Vectored deviation maps for selected prints. Blue colours indicate situations where the scanned images underlie the photo-model and the red colours where the photo-model is slightly elevated. The speckled red reflects the fact that the photo-model resolves individual sand-grains whereas the scan does not. **B.** Mean images for all eight prints one for the scanned images and one for the photo-model showing the subtle differences in print typology that result from the different data capture techniques. Note the colour map is revised here, warm colour indicate areas of maximum depth. **C.** The left hand images is the Statistical Parametric Map (SPM) of t-values produced by a pixel-wise comparison of the two means – photo-model versus scan; warm colours show maximum positive deviation, cool colours negative deviation. The right hand images shows the results when a threshold of T<0.1 is applied with probability values. Given the very low threshold value applied here it is safe to say that there is little statistical difference between the two means. What differences are visible at this low threshold value occur around the longitudinal medial arch and in a proximal position to the toe pads.

To overcome the issue of scaling in this analysis, all subsequent comparisons were conducted on photo-models scaled to the scans and co-registered in Rapidform 2006 first using an initial registration process involving the selection of six or more points on the footprint surface and then using a standard whole surface matching algorithm. Figure 4 shows the maximum shell deviations in the combined model for each of the eight beach footprints; warm colours indicate areas of maximum deviation or model thickness, cool colours indicate minimum areas. Figure 5A shows three prints in which the thickness has been expressed as either a positive or negative vector with the scan surface always being below the photo-model. The maximum deviation over the whole surface can be expressed as a histogram indicating the range of deviation values present (Fig. 3B). Over the vast majority of the surfaces there is little or no deviation, the visible speckling reflects the fact that the photo-models resolve some areas individual sand-grains whereas the scan tends to produce a more uniform surface. Maximum deviation occurs in areas of maximum elevation change, with the scans tending to produce features with crisper edges to features (Fig. 4). In the majority of cases these differences are less than 0.5 mm. For example Print #2 73.2% of the deviation is in the range of ±0.17 mm and only 2.5% greater of the variation is greater than 1 mm.

**Figure 6:**
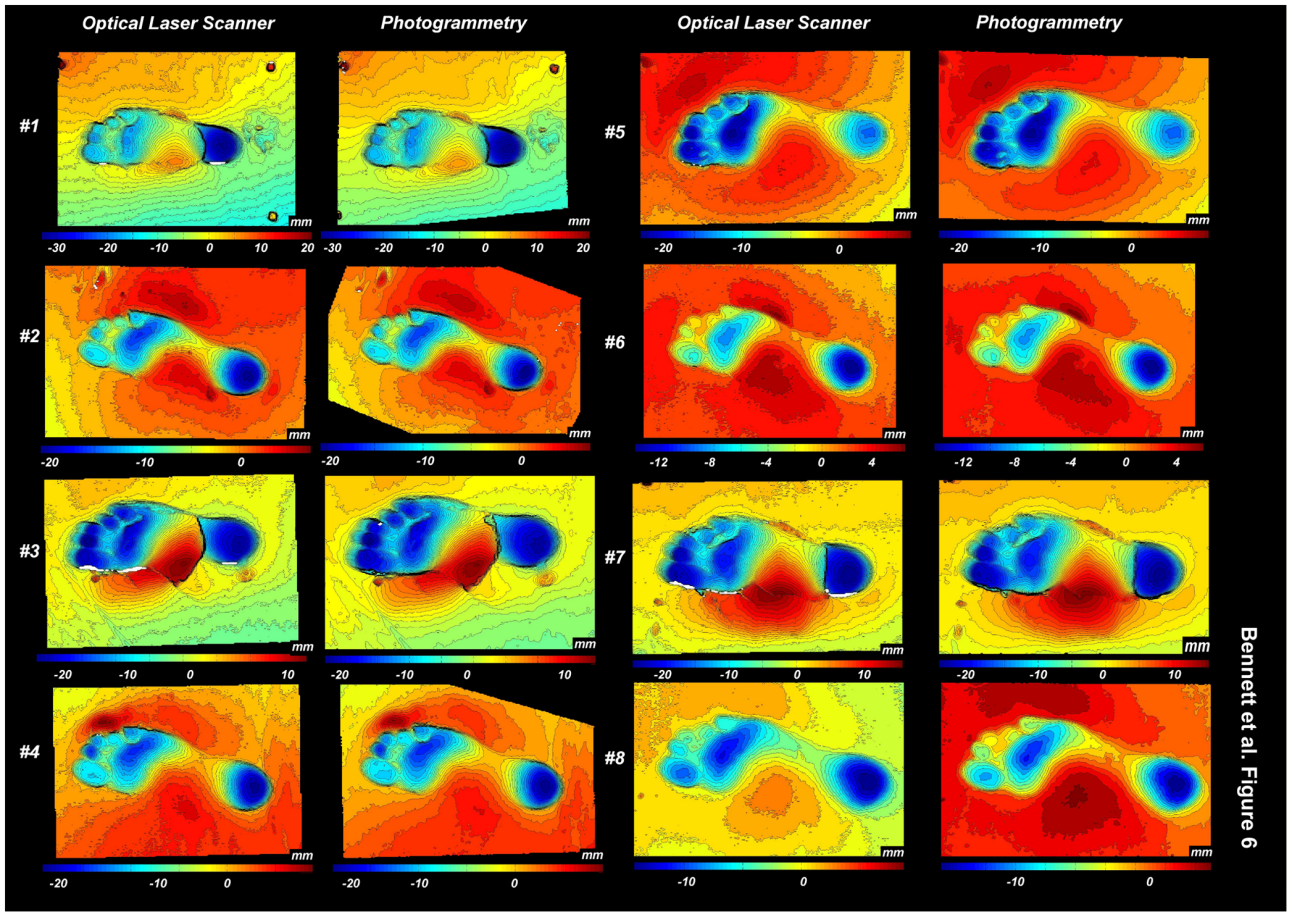
Contour maps for the eight beach prints generated from either the scan or photo-model. Contour interval is 1 mm. Note that the left prints have been inverted to be consistent with the right ones a necessary step in the application of pSPM to the two print populations.

**Figure 7:**
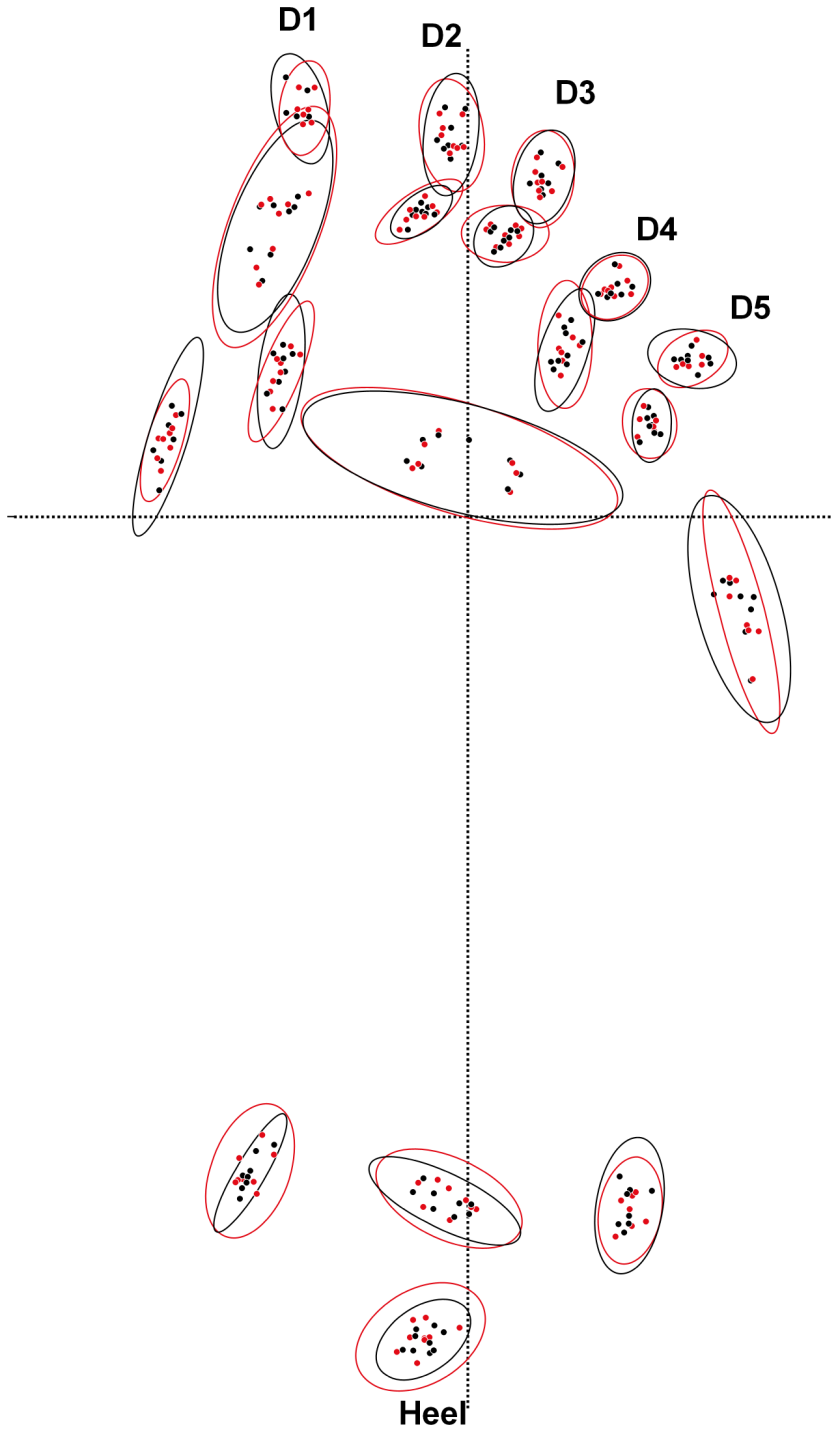
Illustration of a landmark analysis conducted from the scanned and photo-models. Note the almost identical overlap between the 95% probability ellipses.

To examine this further both co-registered surfaces were exported independently as separate asc files and loaded into Foot Processor where 1 mm contour maps where produced from the point cloud data (Fig. 6). Both sets of contour maps are broadly similar, with slight variation to the line smoothness caused by textual variation in the different models and subtle changes to contour extent most notable in Print #8. Edges are more pronounced and sharper on the scans compared to the photo-model consistent the previous observations. Despite these differences broad topographic form and therefore anatomical footprint typology, are similar irrespective of the method used. Landmarks placed on each of these contour maps gives a similar distribution of outputs (Fig. 7).

The recent application of pedobargraphic Statistical Parametric Mapping (pSPM) to footprint studies [2], [49] allows one to calculate a mean footprint from a trackway. Two means were calculated for the same trail using photo-models and scans for each (Fig. 5B). The means shows subtle difference in print typology but using pair-wise t-tests these differences are not statistically significant unless the probability image has a threshold value of T<0.1 applied demonstrating clearly that subtle difference in the derived means do exist but are not statistically significant at normal levels. In conclusion, the two methods do represent very slightly different versions of ‘reality’ however these differences are extremely subtle and are not statistically significant except at the finest of thresholds.

These results do not, however, necessarily hold across all scenarios. Accurate digital photogrammetry is dependent on three key factors: (1) pixel variance caused by textural patterns of the surface; (2) the quality and number of photographs and the range of angles from which they are taken to maximise the potential for triangulation on an identified pixel; and (3) the algorithms used. One of the cast prints was made of plaster and as a consequence has little surface contrast compared to those made out of concrete or taken on the beach. The lack of textural variation impacted the photo-model quality and is illustrated by the large variance recorded (Fig. 3B). Digital photogrammetry is also very dependent on the software used, which is advancing rapidly [48], and the results obtained as illustrated in Figure 3B by using different software options is considerable and needs careful consideration.

Equally scanning is not without faults. One of the criticisms levelled in the past at scanning is that it is dependent in field settings and in particular keeping the scanner stable in windy conditions especially when using scanner models that take multiple or slow passes across the surface. The associated variance cause by this can be examined by comparing multiple shots of the same surface taken over a ten minute interval in windy conditions, which suggests at least for the make and model of scanner used here (Vi900 Konica-Minolta) this is of negligible importance (Fig. 3B).

On the basis of this simple analysis one can suggest that in broad terms there is little to choose between the different methodological approaches and that both give a similar rendition of print typology and that both are subject to field limitations. Issues of scaling need to be addressed carefully when adopting a photogrammetric approach, however, although this is a post-processing issue. These statements however only hold for the equipment, software and surfaces examined here and need to be carefully evaluated by each practitioner on deployment since the number of possible permutations and variables are considerable. It is difficult in practice to say which of these techniques produces a closer representation of ‘reality’ when the differences are in many cases sub-millimetre in scale. Both methods yield accurate data for scientific analysis and both can be used to print three-dimensional models for public visualisation. In the analysis performed here it would appear that the scans are slightly more accurate than the photo-models but in practice this is heavily influenced by logistical issues associated with field deployment.

### Field Deployment

The above analysis provides a context but one of the key determinants of the technique deployed in the field will be the relative logistics involved in using the different approaches. The authors have captured human and animal footprints in a range of different environments [2], [7], [9] including museums, coastal mud and peat flats in the UK, wooded flanks of Italian volcanoes, Namibian sand dunes, margins of playa lakes in Kenya, and semi-arid deserts in Kenya and Central America initially using optical laser scanning and more recently photogrammetry. Based on this extensive field experience we have reviewed the logistical issues that impact on the deployment of either photogrammetry or optical laser scanning (Table 1).

**Table 1:** A summary of the relative merits in terms of field deployment of optical laser scanning versus photogrammetry.

	Photogrammetry	Optical Laser Scanning
**Costs**		
Hardware	Low field costs since a basic digital camera and memory cards are all that is required. Modest lab costs associated with provision of suitable CPU, dependent on the speed of processing required and software to be run; reducing all the time as standard computational power increases.	High depending on the make and model of the scanner used. Low lab costs since no special computational power is required unless a large number of scanned images are being tessellated.
Software	Zero to modest depending on the software used to generate photogrammetric models. Three-dimensional image software required for post processing and visualisation both commercial and freeware options available.	Variable, most expensive scanners come with basic three-dimensional image software required for post processing and basic visualisation less expensive scanners often don’t.
**Deployment**		
Transport logistics	Easy - photo-scale and camera. In some cases use of tripod mounted arms or A-frames may increase the equipment volume.	Depending on scanner model and the support mechanism – tripod or frame - can be quite bulky. Provision of power supply via a converter and a generator, car battery or lithium ion battery.
Electrical Requirements	Minimal, power is required for camera batteries and photo storage devices such as a laptop or PDA.	Most scanners either require a generator, car battery or lithium ion battery with or without a power inverter, either to power the scanner directly, or to recharge a built in battery. Power is also required for PDA or laptop used to run the scanner.
Data capture time	Approximately 5 minutes per print to take between 20 and 30 photographs per print; quicker times possible when using fixed point frames/tripod requiring a more limited number of images. It is possible to have multiple prints or areas being captured simultaneously with multiple photographers. Photographs can also be collected from Unmanned Aerial Vehicles (UAV) especially where large areas are involved, although this may increase the associated costs and logistics.	Depends on the scanner model and resolution required but usually less than 1 minute per scan. Limited to the number of scanners available to one field project.
Post-processing time	Depends on the software being used and the number of images but post-processing time to generate the model can be up to 12 hours, typically 30 to 45 minutes for a high resolution model.	Depends on the tasks being performed and the degree of data cleansing and optimisation required but can be anything from a few minutes to 30 minutes maximum. Aligning multiple scans, especially from long range scanners with high data throughput can take considerable time (up to 24 hours).
Reconnaissance operation and/or training?	Images can be captured by any operator with a digital camera and basic knowledge of photographs required.	Requires access to equipment and basic training.
Memory Requirements	Can be managed by multiple data cards, field based download to laptop or PDA, or field based upload via internet connection. Data volumes are high depending on the individual pictures resolution; for example, one gigabyte for a trail of 10 prints.	Depends on the make and model of scanner, some scanners can record directly to a data card, most required laptop operation. Typical file sizes are between 1 and 5 megabytes per print, though high resolution scans of large areas (e.g. whole or partial track sites) can be many Gb in size.
Risks to site	Damage can be high from feet of photographer taking multiple images from different angles; damage from the feet of tripods or other fixed arm camera mounts. These can be overcome through the use of UAV’s although their use increases costs and logistics	Damage from tripods or scanner frames can be high.
**Accuracy of Outcome**		
Prohibitive environmental conditions	Sunlight & intense shadow can be problematic and shading may be required for the whole area of the print depending on the colour of the substrate and angle of the sun. Wind-blown dust and rain may hinder operation. Wet rock/sediment surfaces or those with residual water content can limit the accuracy of some models especially where it is variable across a surface.	Most high resolution optical scanners require sunlight shading and protection from wind-blown dust and rain. Scanners can fail to operate in very high ambient temperatures due to sensitive components. Air moisture can also cause interference and laser detection issues.
Accuracy and completeness	Dependent on the quality and number of images obtained and the software used to produce the model. Undercut areas can cause problems as can deep prints causing shade problems at the bottom of the print. For accurate measurements images have to be carefully scaled.	Dependent upon the make and model of the scanner. Difficult to capture undercut or overhanging areas with a vertically mounted scanner; multiple shots may be required and there still may be problems with very deep prints. Scans are scaled accurately as they are captured, provided the scanner is regularly calibrated.
Intra- and inter-site variability	The accuracy of a photo-model is specific to one object and the images taken, there is therefore a strong risk of undetected intra- and inter-site variability in accuracy and reliability of the models. The accuracy of every single model needs to be checked via a reference object in every model.	Provided a scanner is well-maintained and regularly calibrated by the manufacturer its accuracy should be consistent in intra-site setting and inter-site settings subject to a caveat around changing environmental conditions. The accuracy of scanned images needs only to be checked once at a site, or following best practice daily at most.
Edge effects	Taking images close to an excavation wall can be problematic since a full 360° array of images may not be possible.	Depends on tripod or frame configuration, but potentially not a problem especially if oblique scans are also used.
Risks of failure	Data quality - moderate to high, associated with failure to capture sufficient images of good quality and coverage especially when post-processing is being done on return from the field. Equipment - low since cameras are ubiquitous on field expeditions so multiple options are often available when one camera fails assuming flexible camera mounts and tripod connections. Post-processing – moderate to high, failure of the software to produce adequate models.	Data quality - low in terms of failure to capture data since the quality of a model can be instantly verified and checked in the field and scans re-shot if needed. Equipment - moderate to high since scanners are relatively delicate scientific equipment and field failure is usually terminal since few projects have access to multiple scanners. This is low for scanners designed for field use. Post-processing – low focused simply on data quality and enhancement.

Photogrammetry offers advantages in the field of being easily deployed with relatively little investment in equipment or complex field logistics. A standard digital camera is all that is required and multiple shots around an image or the use of fixed point photographs from a pre-set tripod rig. Problems may be encountered with deeply impressed prints especially around edge effects as identified in the previous section. Uniform substrate textures, especially under intense sunlight, may limit the accuracy and reliability of some photo-models. Damage to the site may occur due to tripod/frame legs or by standing/crouching on delicate surface in order to take multiple oblique shots. The technique is limited with respect to prints adjacent to excavation walls. While field deployment is relatively fast, cheap and consequently easy one should not neglect the fact that post-processing of high-quality models can be computationally intensive. The principal risk is that the digital elevation models are post-processed and therefore faults are usually determined once a field scientist has left the field. While in many situations one can return to the field this is not always possible if the subject has been lost to erosion or is located in an inaccessible location. Accuracy and reliability may vary between models, since they are determined not by the reliability and consistency of the equipment but the individual combination of photographs used. For precision work therefore every model should be calibrated and checked for accuracy and there may be problems associated with progressive inaccuracy where multiple models are tessellated since they are not necessarily of equal quality in terms of, for example, point cloud density.

In contrast optical laser scanners involve high capital investment, and are more complex to deploy in the field due to power requirements. Most scanners that are designed for engineering or medical purposes have to be protected/mounted within custom-built rigs to allow field deployment, and these may also pose a risk to fragile surfaces. Once deployed a scanner can however give fast, accurate and reliable results across a range of surface textures and right to the edge of an excavation. Data quality and accuracy can be checked in the field and scans re-shot if necessary, minimising risks. Risk of equipment failure is higher given that scanners are relatively delicate scientific equipment. The senior author remembers keenly shorting a scanner in northern Kenya transported at great cost on day one of a field expedition when it was attached to a poorly functioning generator which produced a power spike which exploded the scanner and set the attached laptop alight!

What is clear from Table 1 is that there is not a perfect solution, and field practitioners need to be aware of the rival merits of both optical laser scanning and photogrammetry. Where the highest standards of accuracy and reliability are required either because of a remote location or because the prints will only be exposed in an optimal state once, for example on first excavation, then we would advocate the use of optical laser scanning supplemented by photogrammetry. Where prints are less fragile, more accessible and a greater degree of intra-site variability is acceptable then photogrammetry provides a rapid and flexible solution, particularly ideal for initial recognisance type work. Given that photogrammetry can be accomplished from collections of photographs from multiple cameras, it is worth noting that even when employing optical laser scanners, photogrammetric models may still be produced from field crew’s photographs to compliment planned data collection.

## Discussion and Conclusion

In the first part of this paper we challenged the conventional view that human trackways should be conserved through some form of direct artefact-based preservation strategy especially when of high scientific value. Instead we argued that an approach based on the concept of record and rescue was perhaps more appropriate especially for sites preserved in soft-sediment and therefore easily eroded. In the second part of the paper we explored the tools currently available to capture three-dimensional surfaces – footprints – focusing particularly on evaluating the rival merits of optical laser scanning and digital photogrammetry. The analysis presented here shows that both methods are comparable in broad terms with each having different logistical advantages or disadvantages in the context of field deployment as one might expect. In terms of accuracy and precision of subsequent morphometric measurements scanning is probably on balance more accurate; the scans don’t need to be scaled, have a high and consistent density of points per unit area depending on the make and model of scanner. They give reliable, consistent and repeatable results. In contrast photo-models have to be scaled each time giving a potential variance in terms of both precision and accuracy between individual models and don’t always provide a perfect representation of the surface for example models often lessen sharper edges. There is also an element of risk with photo-models with occasional failure despite consistent methodologies between prints and therefore there is a clear risk in field deployment in remote areas. This can be partly overcome by making low resolution models in the field and adding additional photographs as required covering holes or gaps in the model created. In contrast to scanning, deployment is fast easy and requires little logistical support. Table 1 provides a decision framework in which practitioners can evaluate the options available to them. In broad terms photo-models provide the ideal scenario for rapid field reconnaissance and in particular in situations where prints are found as by-products of other investigations. They also are well adapted to community based field monitoring and recording which could form an essential part of record and rescue type situations when prints may emerge when ‘no scientist’ is watching. It is possible to see both approaches in a complementary rather than competing fashion; one better suited to large scale excavations with large logistical resource where maximum accuracy is required – such as at Ileret – whereas photogrammetry may be more suited to small scale opportunistic ventures and chance encounters. No doubt this balance will change over time as technology changes and with the increased sophistication of freeware which is driving the development of photogrammetry one suspects that the balance will shift progressively in its favour over the next few years.

A conservation strategy based on the premise that the actual artefact can be lost, provided that the data is captured digitally in three-dimensions irrespective of a sites’ antiquity or palaeoanthropological significance carries with it a number of implications which are worth further exploration. There are two clear elements to a successful strategy of this sort:

1. Site Monitoring. Regular monitoring of a site, especially around predictable/seasonal geomorphological episodes such as seasonal storms when erosion and/or fresh exposure is to be anticipated is vital if a rescue and record strategy is to function well. Sites in densely populated and developed countries this is feasible via either amateurs or site custodians/owners and amateur photogrammetry may have an important role to play in such programmes. For example, on the Sefton Coast the site custodians (National Trust) and the volunteers and amateur enthusiasts which help provide its work force are easily mobilised to provide a monitoring network with access to skilled excavators at a range of local University and Museums should something of particular note emerge. Equally in an area such the Namib Sand Sea geotourism has a role in monitoring sites and bring new discoveries to the attention of the scientific community. In less densely populated countries or where sites are remote and especially in less well developed regions the provision of such regular monitoring becomes much more difficult and challenging. Here monitoring strategies are more likely to be dependent regular visits by teams of excavators interested in a range of targets not just hominin footprints as a result awareness of the potential for human footprints and their recognition in for example vertical section as well as on a surface becomes critical as does the training of such excavators to deal with chance footprint finds. The increased awareness of routine digital photogrammetry within the tool kit of both prospectors and excavators is a key issue here.
2. Site Documentation and Dissemination. For such a strategy to work new footprint exposures need to be accurately document and as argued here this should involve the accurate and precise recording of footprints using appropriate technology to create a realistic three-dimensional representation. This should of course combine with a programme of wider site description and documentation which should potentially involve the archiving of key samples against future scientific requirements or the creation of specific and/or protected via deep burial of an excavation reserve against future advance in technology for sediment description and analysis. The open dissemination of such information to the widest possible set of stakeholders – both scientists, not just those that found the prints, but to all, as well as to the general public and to local stakeholders who have a vested interest in the site – is essential and becomes a critical priority. This is an exercise in both scientific and public engagement coupled with the long-term archiving of data in agreed formats which are able to stand time and the pace of technological innovation and change.

It is this last point which is particularly relevant given the current debate around Open Access Data [50] which is the point that we would like to emphasize. The community needs to agree file formats that are timeless, agree standard archiving protocols and openly share data as part of conservation strategies. As a community this is the challenge that we need to accept and face; how do we share openly, quickly and for all parties – scientific and none - data on human trace fossils irrespective of who excavated or found the prints?
